# Draft genome sequences of two β-glucuronidase positive strains of *Salmonella enterica* subspecies *salamae* isolated from reptile feces in KwaZulu-Natal, South Africa

**DOI:** 10.1128/mra.01301-23

**Published:** 2024-03-11

**Authors:** Maike Claussen, Stefan Schmidt

**Affiliations:** 1 Discipline of Microbiology, School of Life Sciences, University of KwaZulu-Natal, Pietermaritzburg, KwaZulu-Natal, South Africa; Rochester Institute of Technology, Rochester, New York, USA

**Keywords:** *Salmonella enterica *subspecies *salamae*, draft genomes, β-glucuronidase, reptiles, South Africa

## Abstract

Two *Salmonella enterica* isolates obtained from reptile feces displayed β-glucuronidase activity. Nearly complete genome sequences were obtained after shotgun sequencing and *de novo* genome assembly. By comparison to reference genomes, both isolates were identified as *Salmonella enterica* subspecies *salamae* with the sequence type identified as 1208 and the serotype as 42:r:-.

## ANNOUNCEMENT

β-Glucuronidase activity is typically present in *Salmonella enterica* subspecies *diarizonae* but can also occur in the subspecies *enterica*, *salamae*, and *indica* (1). Although human salmonellosis’ is mostly caused by *Salmonella enterica* subspecies *enterica*, infections by other non-*enterica* subspecies do occur but are usually rare (2–6). *Salmonella enterica* subspecies *salamae*, like all non-*enterica* subspecies, are typically associated with cold-blooded animals such as reptiles but can also be found in warm-blooded animals (3, 6–9).

The two *Salmonella* isolates, designated A199 and G77, were originally obtained from agama (2019) and gecko feces (2021). Fecal reptile pellets collected in a suburban garden in Pietermaritzburg, South Africa, were decimally diluted in sterile saline (0.85%). Subsamples of agama fecal dilutions (100 µL) were directly spread-plated onto XLD-Agar (24 h, 37°C). A portion of the pooled gecko feces dilution was pre-enriched in buffered peptone water (24 h, 37°C), followed by selective enrichment in Rappaport-Vassiliadis broth (41.5°C, 24 h) and spread-plating on XLD-Agar (37°C, 24 h). A purified (Nutrient agar) single colony was sub-cultivated in Nutrient broth to prepare glycerol stock cultures for storage at −20°C. Both isolates were confirmed using TSI- and Urea-Agar and by PCR-based detection of the *invA* gene (10). Sanger sequencing (CAF, Stellenbosch) of the 16S rRNA gene amplicon (primers fD1 + rP2) (11) further confirmed that both isolates belonged to the species *Salmonella enterica* (accession no. ON630378.1, ON630388.1). Notably, both isolates showed untypical colony morphology on improved Salmonella-Chromo-Select-Agar due to their β-glucuronidase activity, which was confirmed using TBX agar.

Washed cells of pure overnight cultures of A199 and G77 were transferred into DNA/RNA Shield Lysis Tubes (ZYMO). Extracted DNA (ZymoBIOMICS-96 MagBead kit) was used for shotgun genome sequencing (paired-end 2 × 150 bp, Illumina Nova Seq) by Zymo Research (Irvine, CA, USA) after creating a library (Nextera DNA Flex Library Prep Kit). Using the BV-BRC genome analysis pipeline (12), raw reads were quality filtered, trimmed (Trim Galore v0.6.5, Cutadapt v2.2), assembled (Unicycler v0.4.8, minimum contig size 500 bp), and annotated (RASTtk) (13–16). Default parameters were used for all bioinformatic tools unless otherwise stated. Resistance genes were detected using Resfinder (v.4.1) (17), prophages using Phaster (18), plasmids using Plasmidfinder (v2.1, https://cge.cbs.dtu.dk), and human pathogenicity was predicted using Pathogenfinder (v1.1) (19).

**Fig 1:**
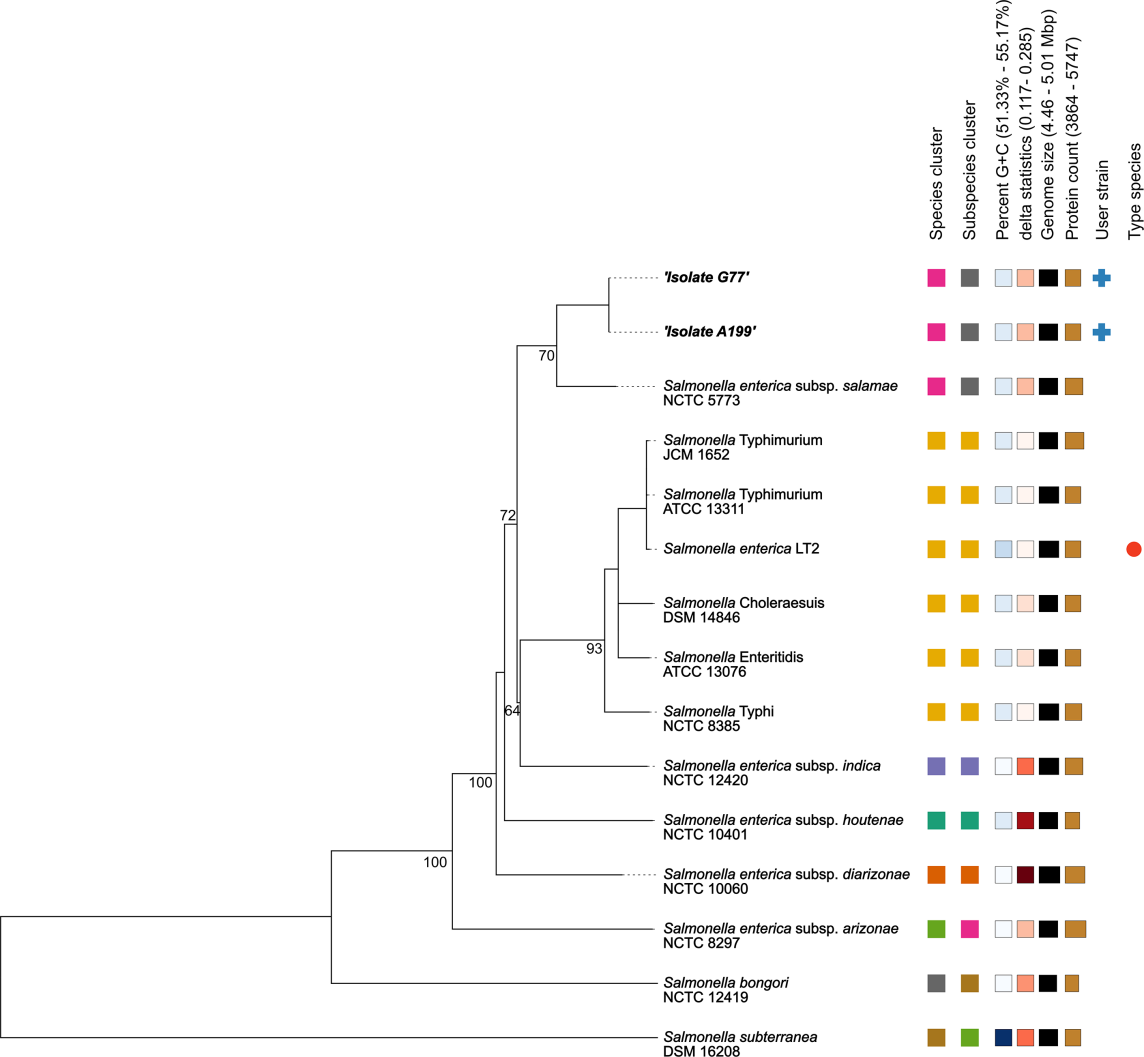
Midpoint-rooted phylogenetic tree inferred with FastME (2.1.6.1) from GBDP distances calculated from type strain genome sequences using the TYGS server. Branch lengths are scaled based on the GBDP distance formula d5; numbers shown are GBDP pseudo-bootstrap support values > 60 % (100 replications, average branch support = 63%).

The results for the main genomic features of both isolates are summarized in Table 1. The genome assembly was estimated to be 97.97% complete using the universal single-copy ortholog benchmarking tool available at http://cab.cc.spbu.ru (20). Using ANIb (21) and the phylogenetic analysis tools available in TYGS (22) demonstrated that the two isolates (G77, A199) belonged to *Salmonella enterica* subspecies *salamae* (Fig. 1). Multilocus sequence typing (MLST v2.0) (23) identified both isolates as sequence type 1208, and the serotype was predicted as 42:r:- (SeqSero2) (24).

**TABLE 1:** Genomic characteristics of two β-glucuronidase positive *Salmonella enterica* subsp. *salamae* strains isolated from reptile feces in South Africa

Strain	A199	G77
Origin, year	Agama feces, 2019	Gecko feces, 2021
Location	Pietermaritzburg, South Africa (29°36'S 30°23'E)	Pietermaritzburg, South Africa (29°36'S 30°23'E)
Identity	*Salmonella enterica* subsp. *salamae*	*Salmonella enterica* subsp. *salamae*
Digital DNA:DNA hybridization (dDDH, d4) best type strain genome match	*Salmonella enterica* subsp. *salamae* NCTC 5773^T^ (83.2%)	*Salmonella enterica* subsp. *salamae* NCTC 5773^T^ (83.2%)
Average nucleotide identity (ANIb) best type strain genome match	*Salmonella enterica* subsp. *salamae* CCUG 30039^T^ (97.73%)	*Salmonella enterica* subsp. *salamae* CCUG 30039^T^ (97.66%)
Predicted serotype	42:r:-	42:r:-
MLST sequence type	1208	1208
Total genome size	4,733,636 bp	4,733,809 bp
Completeness (BUSCO)	97.97%	97.97%
Number of reads (input)	10,679,960	7,179,091
Average read length	140	137
Number of contigs	59	59
N50	226,585	226,585
Largest contig	417,545	417,720
GC content	51.96%	51.96%
Protein coding sequences (CDS)	4,715	4,716
tRNA	73	73
rRNA	3	3
Predicted resistance genes	aac(6′)-Iaa	aac(6′)-Iaa
Salmonella pathogenicity islands	SESS-LEE, SPI-1, SPI-2, SPI-3, SPI-9	SESS-LEE, SPI-1, SPI-2, SPI-3, SPI-9
Human pathogen probability	0.926	0.926
Number of predicted intact prophages	1	1
Plasmids	Not detected	Not detected

Our results show that *Salmonella enterica* subspecies *salamae* strains of the same serotype (42:r:-) are prevalent in the feces of geckos and agama lizards in South Africa. As both *Salmonella* isolates showed a high probability of human pathogenicity, feces shed by these reptiles might be a source of *Salmonella* contamination and a potential health risk, especially for the most vulnerable YOPI (young-old-pregnant-immunocompromised) citizens.

## Data Availability

This Whole-Genome Shotgun project has been deposited at GenBank under the accession number JASMSI000000000 and JASMSH000000000. The version described in this paper is the first version, JASMSI000000000 and JASMSH000000000. The raw sequence reads were deposited under NCBI BioProject accession number PRJNA844906 and NCBI SRA data accession numbers SRR19743658 and SRR19523434.
